# The Asymmetry of White Matter Hyperintensity Burden Between Hemispheres Is Associated With Intracranial Atherosclerotic Plaque Enhancement Grade

**DOI:** 10.3389/fnagi.2020.00163

**Published:** 2020-06-23

**Authors:** Ling Ni, Fei Zhou, Zhao Qing, Xin Zhang, Ming Li, Bin Zhu, Bing Zhang, Yun Xu

**Affiliations:** ^1^Department of Neurology, Affiliated Drum Tower Hospital, Medical School of Nanjing University, Nanjing, China; ^2^Department of Radiology, Affiliated Drum Tower Hospital, Medical School of Nanjing University, Nanjing, China; ^3^Institute of Brain Science, Nanjing University, Nanjing, China

**Keywords:** white matter hyperintensities, intracranial atherosclerotic stenosis, vessel wall plaque enhancement, asymmetry of WMH burden, hypoperfusion

## Abstract

**Purpose:**

The contribution of intracranial atherosclerotic stenosis (ICAS) to the development of white matter hyperintensities (WMHs) has not been fully elucidated. We aimed to retrospectively assess the relationship between WMH burden and unilateral ICAS by combined examination of lumen stenosis, plaque enhancement, and cerebral perfusion.

**Materials and methods:**

A cross-sectional study of 41 patients with symptomatic unilateral ICAS (mean age 57 ± 10 years; 26 males) was conducted. Detailed clinical data, including vascular risk factors, were obtained. WMH volume was derived from 3D-fluid-attenuated inversion recovery (3D-FLAIR) and was assessed by using a validated semi-automated protocol. Lumen stenosis, plaque enhancement, and cerebral perfusion (assessed on time-to-peak parameter using the Alberta Stroke Program Early CT score (TTP-ASPECTS) scale) were evaluated. The WMH volumes of peri-ventricular (PWMH) and deep (DWMH) white matter were calculated separately and compared between hemispheres. Associations between WMH volume (inter-hemispheric volume difference, ipsilateral and contralateral to the ICAS site separately), unilateral ICAS imaging metrics, and vascular risk factors were assessed by using linear regression.

**Results:**

The DWMH volume ipsilateral to the ICAS site (ipsilateral DWMH volume) was significantly greater than that of the contralateral site (*P* < 0.001), while the PWMH volume difference between hemispheres did not reach statistical significance. The inter-hemispheric DWMH volume difference was significantly associated with a higher plaque enhancement grade (β = 0.436, *P* = 0.005) and inversely associated with cerebral hypoperfusion (lower TTP-ASPECTS) (β = −0.613, *P* < 0.001). In the between-subject multivariable regression analysis, while older age (β = 0.323, *P* = 0.025), hypoperfusion (β = −0.394, *P* = 0.007), and hypertension (β = 0.378, *P* = 0.011) were independently associated with ipsilateral DWMH volume, plaque enhancement did not show an association with ipsilateral DWMH volume. The association between ipsilateral DWMH volume and lumen stenosis approached statistical significance (β = 0.274, *P* = 0.084).

**Conclusion:**

The DWMH was attributed to chronic hypoperfusion secondary to atherosclerotic stenosis. The association between the asymmetry of deep white matter lesions and plaque enhancement might suggest that increased deep white matter lesions are those ischemic lesions, which are more prone to the development of stroke.

## Introduction

White matter hyperintensities (WMHs) are a common neuroradiological finding that appear as deep and peri-ventricular white matter lesions with increased signal intensity on T2-weighted imaging with fluid-attenuated inversion recovery (FLAIR). They are highly prevalent among elderly individuals, particularly in patients with symptomatic cerebrovascular disease and known vascular risk factors (Prins and Scheltens, 2015; Lin et al., 2017). WMHs have been associated with infarct growth, cerebral hemorrhage after treatment with tissue plasminogen activator, poor stroke functional outcomes, and an increased risk of stroke recurrence (Fierini et al., 2017; Ryu et al., 2017; Wang S. et al., 2017); thus, a better understanding of the underlying pathophysiology may contribute to the proposal and evaluation of strategies for prevention and treatment.

Large-artery atherosclerosis has been suggested to contribute to the development of WMHs. However, to date, no definitive correlation between white matter burden and cerebral atherosclerosis has been found based on the existing literature (Hajdarevic et al., 2016; Kwon et al., 2016; Lucatelli et al., 2016; Ammirati et al., 2017; Rundek et al., 2017). Given the overall prevalence of extracranial carotid stenosis (ECS) in Caucasian populations and the higher frequency of intracranial atherosclerotic stenosis (ICAS) in Asian individuals (Pu et al., 2013; Liu and Chen, 2014), it is important to investigate the relationship between ICAS and WMHs. Unlike the ECS, the collateral branches across the circle of Willis that usually compensate for perfusion in the distal region of the occlusion, patients with ICAS have only meningeal to pial or pial collaterals that are perfused. As a result, ICAS patients seem to be more prone to perfusion deficits. However, information concerning the role of ICAS in WMH is limited (Kim et al., 2014; Park et al., 2015; Kwon et al., 2016; Nam et al., 2017). In addition, most researches have been based on whole-brain WMH evaluation with an assumption of symmetry. Only a small number of studies have investigated the WMH burden in the cerebral hemisphere ipsilateral to carotid artery stenosis, and inconsistent results have been reported (Baradaran et al., 2017), which may be ascribed to the reasons mentioned above. Therefore, multiparameter studies combining the lumen, plaque, and perfusion assessment of unilateral ICAS patients would provide more precise information on the relationship between the WMH burden and atherosclerosis.

To our knowledge, the relationship between WMHs and unilateral ICAS in symptomatic patients has not yet been investigated. Thus, our present study aimed to assess the relationship between WMHs and unilateral ICAS by combined examination of lumen stenosis, plaque enhancement, and cerebral perfusion. Our hypotheses are as follows: (1) a unilateral ICAS would increase the burden of ipsilateral WMH volume, and (2) plaque enhancement and cerebral perfusion might contribute to the burden of WMHs.

## Materials and Methods

### Subjects

**FIGURE 1:**
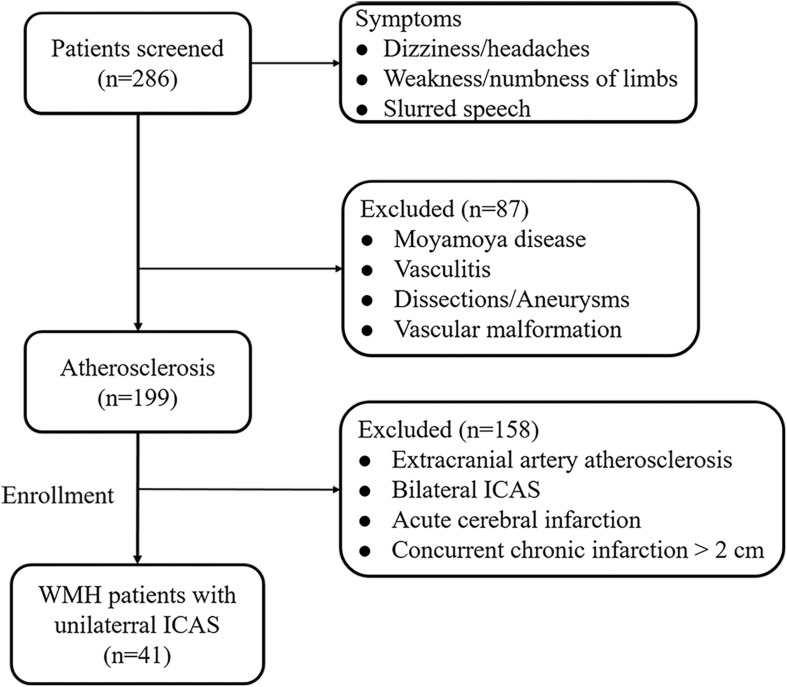
Flowchart for patient inclusion.

A total of 286 subjects with dizziness, headaches, weakness/numbness of the limbs, or slurred speech were initially referred to the Department of Neurology, Affiliated Drum Tower Hospital, Medical School of Nanjing University, between July 2015 and September 2016. Intracranial and extracranial vascular examinations, including high-resolution (HR-MRI) and three-dimensional-FLAIR (3D-FLAIR) images, were screened by two experienced neuroradiologists (FZ and ZQ), and subjects were included in the study according to the following criteria: (a) unilateral ICAS regardless of the degree of stenosis and without concomitant lesions (plaques and stenosis) in the ipsilateral EC and contralateral intracranial/extracranial arteries, determined by high-resolution (HR-MRI) vessel evaluation; (b) imaging evidence of normal performance on diffusion-weighted imaging (DWI) to avoid confounding WMHs with acute cerebral infarction; and (c) MRI white matter disease burden ≥ Fazekas grade 1. The exclusion criteria were as follows: (a) non-leucoaraiosis-related white matter lesions (e.g., multiple sclerosis); (b) non-arteriosclerotic intracranial artery stenosis related to dissection, Moyamoya disease, etc.; (c) any ischemic or hemorrhagic infarction or concurrent chronic infarction >2 cm; (d) a contraindication to MRI; or (e) other morphologic brain abnormalities other than vascular lesions, such as tumors. According to the above criteria, 87 subjects were excluded due to Moyamoya disease, vasculitis, dissections/aneurysms, or vascular malformation. For the remaining 199 atherosclerosis subjects, 158 subjects were excluded due to extracranial artery atherosclerosis, bilateral ICAS, acute cerebral infarction, or concurrent chronic infarction >2 cm. Finally, 41 WMH subjects with unilateral ICAS were enrolled in the study (Figure 1). All of the subjects were treated only with conventional management, such as antiplatelet, lipid-lowering, and neuroprotective agents but not with intravenous thrombolysis or endovascular therapy before MRI acquisition. Written informed consents were acquired from all patients or their guardians, and our study was approved by the Drum Tower Hospital Research Ethics Committee.

### Vascular Risk Factor Assessment

Clinical information included age, sex, history of hypertension (systolic blood pressure >140 mm Hg and/or diastolic blood pressure >90 mm Hg or is using antihypertensive drugs), diabetes mellitus (fasting serum glucose level of 7.0 mmol/L or non-fasting serum glucose level of 11.1 mmol/L or is treated for diabetes mellitus), and dyslipidemia (total cholesterol level >200 mg/dl or low-density lipoprotein cholesterol >130 mg/dl or is using lipid-lowering drugs). Also, a history of smoking and previous cerebral ischemic events was recorded.

### MRI Data Acquisition

MRI scanning was performed on a 3-Tesla MR scanner (Achieva 3.0T TX dual Medical Systems; Philips Medical Systems, Eindhoven, Netherlands) with an eight-channel phased array head coil. The MR scanning protocol included DWI, 3D-FLAIR, T_1_WI-volumetric isotropic turbo spin-echo acquisition (T_1_VISTA), dynamic susceptibility contrast perfusion-weighted imaging (DSC-PWI), and T_1_VISTA C+. T_1_VISTA in the coronal plane for intracranial vessel wall imaging was performed using the following parameters: TR/TE, 800/19 ms; FOV, 200 mm × 180 mm × 40 mm; matrix size, 324 × 292; and acquisition time 6 min. Post-contrast T_1_VISTA images were acquired 5 min after intravenous injection of gadodiamide (Gd-DTPA-BMA, Nycomed) at a dose of 0.1 mmol/kg. 3D-FLAIR was performed using the following parameters: TR/TE, 4800/282 ms; FOV 250 mm × 250 mm × 180 mm; matrix size 224 × 224; and acquisition time 4 min 52 s. DSC-PWI was performed using a gradient-echo echo planar imaging (EPI) sequence: TR/TE, 2000/30; matrix size, 96 × 95; FOV, 224 mm × 224 mm; flip angle, 90°; and slice thickness, 4.0 mm. DWI images were obtained using the following parameters: *B*_max_, 1,000 s/mm^2^; TR/TE, 2200/56 ms; matrix, 192 × 109; field of view (FOV), 230 mm × 220 mm; flip angle (FA), 90°; and slice thickness, 6.0 mm.

### WMH Volume Quantification

To help distinguish the acute infarcts from WMHs, we checked the DWI, apparent diffusion coefficient (ADC), and 3D-FLAIR sequences. WMHs were classified as deep (DWMHs) and peri-ventricular (PWMHs) separately for both hemispheres. Segmentation and volume quantification of WMH was performed on 3D FLAIR images using the MRIcro software (University of Nottingham School of Psychology, Nottingham, United Kingdom)^1^ and ITK-SNAP^2^ (Yushkevich et al., 2006). We briefly describe the process as follows: (a) regions of interest (ROIs) representing WMHs were outlined in the regions of the DWMH and PWMH bilaterally; (2) an intensity filter was applied to 3D-FLAIR images; (3) the intersection between the initial ROI and the intensity filter was checked by an experienced assessor to generate the final ROI; and (4) the WMH volume calculations were finalized after adjusting for head size by using the intracranial area. The WMHV was expressed as percent total intracranial volume (ICV) [100^∗^(WMH/ICV) = WMHV] to correct for head size and was log-transformed to approach a normal distribution.

### Intracranial Vessel Wall Plaque and Lumen Evaluation

The intracranial vessel wall images were evaluated by two independent neuroradiologists (LN, FZ, with 7 and 4 years of experience in neurovascular imaging, respectively), who were blinded to the WMH information, using a Philips MR workstation (Extended Workstation, EWS) (Qiao et al., 2014). The range of intracranial atherosclerosis we evaluated included the intracranial portion of the internal carotid artery (ICA) to the second segment of the anterior, middle, and posterior cerebral arteries. The degree of plaque enhancement was graded as follows: 0 = no enhancement; 1 = mild enhancement; and 2 = obvious enhancement (Qiao et al., 2014). The presence or absence of contrast-enhanced lesions in the aforementioned arteries was determined. If multiple arterial segments showed plaque enhancement, the segment with the most severe stenosis was considered the location of the lesion. The ICAS stenosis was graded as follows: 1 = mild (<50%); 2 = moderate (50–69%); 3/4 = severe stenosis/occlusion (70–100%), based on the North American Symptomatic Carotid Endarterectomy Trial Collaborators (NASCET) criteria.

### Cerebral Perfusion Evaluation

Perfusion data were analyzed using a Philips advanced workstation. After selection the arterial input function (AIF) and circular singular value decomposition of the concentration time curve, time-to-peak (TTP) maps were generated to indicate tissue at risk (Zaro-Weber et al., 2010). Two neuroradiologists who were blinded to other information evaluated the cerebral perfusion based on TTP maps, according to the Alberta Stroke Program Early CT score (ASPECTS). Each ASPECTS area was scored 1 if normal and 0 if abnormal. Finally, we added these sub-scores to calculate the final TTP-ASPECTS for each patient (range 0 to 10).

For any discrepancy about the plaque enhancement, lumen stenosis, and TTP-ASPECTS, another senior neuroradiologist (XZ with 15 years of experience) reevaluated the images and helped reach consensus.

### Statistical Analysis

All continuous numerical variables were expressed as the mean ± SD, with the exception of WMH volume, which was expressed as the median (Quartile 1–Quartile 3). The categorical variables are presented as frequencies. The paired Wilcoxon signed-rank test was used to compare volumes of DWMHs, PWMHs, and total white matter hyperintensities (TWMHs) ipsilateral and contralateral to the ICAS site.

To assess the independent predictors of the inter-hemispheric WMH volume differences, lumen stenosis grade, plaque enhancement, TTP-ASPECTS, and location of plaque were entered into the multivariate linear regression model, as the clinical information and vascular risk factors were matched by using a within-patient design.

The relationships among WMH volume (DWMH and PWMH, both ipsilateral and contralateral to the ICAS site), vascular risk factors, and ICAS imaging metrics (plaque enhancement, TTP-ASPECTS, lumen stenosis, and location of plaque) were assessed in separate linear regression analyses. Any of these variables that were individually associated with WMH volume in the univariable analyses with a *P*-value < 0.1 were selected into the multivariable models.

Spearman rank correlation was used to analyze the relationship between the ICAS imaging metrics and WMH volume, and scatter plots were generated. Differences in WMH volume between patients with different stenosis grades and different plaque enhancement grades and between those with and without hypertension were evaluated using a two-sample *t*-test and ANOVA and by generating bar graphs.

The inter-rater reproducibility for grading plaque enhancement and lumen stenosis was evaluated using Cohen’s kappa analysis. The inter-rater reproducibility for the assessment of TTP-ASPECTS was evaluated using the intraclass correlation coefficient (ICC). The reliability of <0.4 was considered poor, the reliability of 0.4–0.75 was considered good, and the reliability of >0.75 was considered excellent. All statistical analyses were conducted using SPSS for Windows version 22.0 (SPSS Inc., Chicago, IL, United States), and a *P*-value < 0.05 was considered statistically significant.

## Results

### Clinical Characteristics of Patients

Table 1 illustrates the demographic features, vascular risk factors, and imaging metrics of the included patients. The mean age of the 41 patients enrolled was 57 ± 10 years; 36.6% were women. Hypertension (63%) and hyperlipidemia (26.8%) were the main vascular risk factors in this sample, followed by smoking and diabetes (both 19.5%). Among the 41 patients, more than half showed strong enhancement (grade 2) of the vessel wall plaque as well as high-grade stenosis/occlusion (both were 56.1%), 80.5% of the vulnerable plaques are distributed in middle cerebral artery, and 63% showed compromised cerebral perfusion with ASPECTS ranging from 2 to 10.

**TABLE 1:** Clinical and imaging characteristics of the unilateral intracranial atherosclerosis patients (*n* = 41).

**Demographics**	**Value**
Age, year, mean ± SD, (range)	57 ± 10.5, (38–77)
Men, no. [%]	26 [63]
**Vascular risk factors, no. [%]**	
Hypertension	26 [63]
Diabetes mellitus	8 [19.5]
Hypercholesterolemia	11 [26.8]
Current smoking	8 [19.5]
**Imaging metrics**	
Plaque enhancement, no. [%]	
Grade 0	8 [19.5]
Grade 1	10 [24.4]
Grade 2	23 [56.1]
**Degree of stenosis, no. [%]**	
Grade 1	10 [24.4]
Grade 2	8 [19.5]
Grade 3/4	23 [56.1]
**Location of vulnerable plaque**	
ICA	6 [14.6]
MCA	33 [80.5]
ACA	2 [4.9]
**Cerebral perfusion**	
Hypoperfusion, no. [%]	26 [63]
ASPECTS range	2–10

*ASPECTS, Alberta Stroke Program Early CT score; SD, standard deviation; no., number.*

### WMH Volume Comparison and the Relationship With Intracranial Atherosclerosis and Vascular Risk Factors

The volumes of WMHs in the DWMH, PWMH, and TWMH, both ipsilateral and contralateral to the ICAS site, are shown in Table 2. The DWMH volume ipsilateral to the ICAS site (ipsilateral DWMH volume) was significantly higher than that of the contralateral site (median, interquartile range (IQR), 240.91 mm^3^, 645.09 mm^3^ vs. 85.20 mm^3^, 368.92 mm^3^; *P* < 0.001), while the PWMH volume difference did not reach statistical significance (median, IQR, 5 mm^3^, 121.5 mm^3^ vs. 5 mm^3^, 106.5 mm^3^; *P* = 0.469).

**TABLE 2:** WMH volume ipsilateral and contralateral to the intracranial atherosclerosis site.

	**Ipsilateral**	**Contralateral**	***Z***	***P***
DWMH, mm^3^ median (Q1–Q3)	240.91(37.66−682.75)	85.20(14.68−383.60)	−4.010	**<0.001**
PWMH, mm^3^ median (Q1–Q3)	5(5−126.50)	5(5−111.50)	−0.724	0.469
Total, mm^3^ median (Q1–Q3)	279.00(37.98−1283.14)	85.20(14.68−554.85)	−3.168	**0.002**

*DWMH, deep white matter hyperintensities; PWMH, peri-ventricular white matter hyperintensities. Total means the sum of white matter volumes of the deep and peri-ventricular WMHs from the same site. Q means quartile. The bold font indicates that the difference achieved statistical significance.*

**FIGURE 2:**
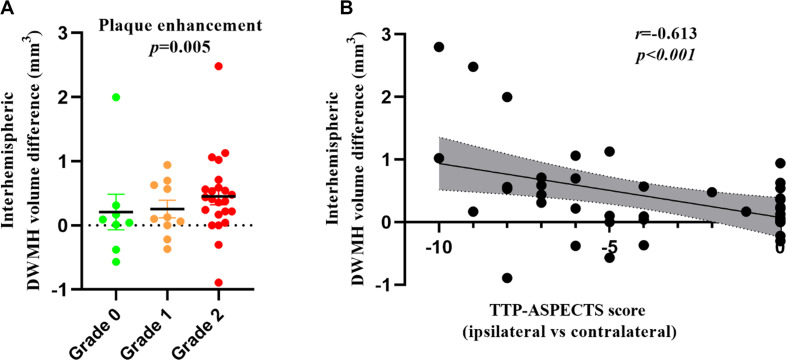
Bar graphs showing the inter-hemispheric DWMH volume difference among patients with different plaque enhancement grades **(A)**. Scatter plots showing the relationship between TTP-ASPECTS and the inter-hemispheric DWMH volume difference **(B)**. WMH volume was log-transformed to normalize the distribution.

The results of linear regression of unilateral ICAS imaging metrics with the inter-hemispheric WMH volume differences are listed in Table 3. The inter-hemispheric DWMH volume difference was significantly associated with plaque enhancement grade (β = 0.436, *P* = 0.005) and inversely associated with TTP-ASPECTS (β = −0.613, *P* < 0.001) but not with the degree of lumen stenosis (β = −0.243, *P* = *0.146*). No association was found between inter-hemispheric PWMH volume difference and the three abovementioned imaging metrics (all *P* > 0.05). In Figure 2, scatter plots show the relation between the inter-hemispheric DWMH volume difference and TTP-ASPECTS. The bar graphs show the relationship between the inter-hemispheric DWMH volume difference and different plaque enhancement grades.

**TABLE 3:** Associations between imaging parameters and inter-hemispheric DWMH volume difference.

**Model**	**Standardization coefficients**	***t***	**Sig.**
	**Beta**		
(Constant)		5.212	0.000
Lumen stenosis	−0.243	−1.496	0.146
Plaque enhancement	0.436	3.017	**0.005**
ASPECTS score	−0.6 wode 13	−3.992	**0.000**

*DWMH, deep white matter hyperintensities; ASPECTS, Alberta Stroke Program Early CT score. The bold font indicates that the difference achieved statistical significance.*

**FIGURE 3:**
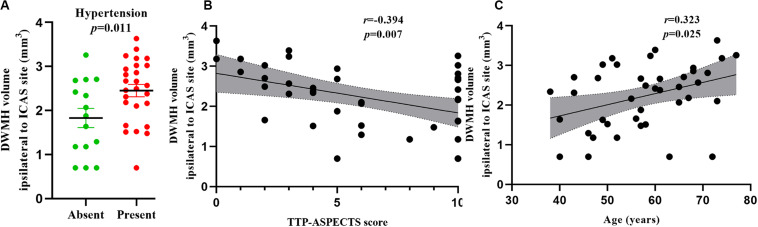
Bar graphs showing the relationship between DWMH volume ipsilateral to the ICAS site and the status of hypertension **(A)**. Scatter plots showing the relationship between DWMH volume ipsilateral to the ICAS site and TTP-ASPECTS **(B)** and age **(C)**. WMH volume was log-transformed to normalize the distribution.

In univariate analysis, age (β = 0.364, *P* = 0.019), hypertension (β = 0.375, *P* = 0.016), lumen stenosis (β = 0.329, *P* = 0.035), and TTP-ASPECTS (β = −0.428, *P* = 0.005) were significantly associated with ipsilateral DWMH volume with a *P*-value < 0.1 and therefore were included as covariates in the subsequent multivariate analysis. There was no statistical association between ipsilateral DWMH volume and plaque enhancement grade (β = 0.095, *P* = 0.556). In the multivariate analysis, only age (β = 0.323, *P* = 0.025), hypertension (β = 0.378, *P* = 0.011), and TTP-ASPECTS (β = −0.394, *P* = 0.007) remained significantly associated with the ipsilateral DWMH volume. Age (β = 0.544, *P* < 0.001) was significantly associated with PWMH volume ipsilateral to the ICAS site. Age (β = 0.428, *P* = 0.003) and TTP-ASPECTS (β = −0.343, *P* = 0.014) were significantly associated with TWMH volume ipsilateral to the ICAS site. The association between ipsilateral DWMH volume and lumen stenosis approached statistical significance (β = 0.274, *P* = 0.084). The results are shown in Tables 4, 5. In Figure 3, scatter plots show the relationship among the ipsilateral DWMH volume, age, and TTP-ASPECTS. The bar graphs show the relationship between ipsilateral DWMH volume and the status of lumen stenosis, plaque enhancement, and hypertension.

**TABLE 4:** Linear regression analysis of the relationship between DWMH volume ipsilateral to ICAS site and demographic, clinical, and radiological factors.

**Model**	**Standardization coefficients**	***t***	**Sig.**
	**Beta**		
**Univariate model**			
Lumen stenosis	0.329	2.179	**0.035**
Plaque enhancement	0.095	0.593	0.556
ASPECTS	−0.428	−2.955	**0.005**
Age	0.364	2.442	**0.019**
Hypertension	0.375	2.524	**0.016**
Smoking	−0.036	−0.224	0.824
Hyperlipidemia	−0.208	−1.330	0.191
Diabetes mellitus	0.149	0.940	0.353
**Multivariate model**			
(Constant)		0.693	0.492
ASPECTS	−0.394	−2.855	**0.007**
Age	0.323	2.340	**0.025**
Hypertension	0.378	2.940	**0.011**
Lumen stenosis	0.274	1.776	0.084*

DWMH, deep white matter hyperintensities; ASPECTS, Alberta Stroke Program Early CT score. The bold font indicates that the difference achieved statistical significance. *Indicates that the difference approached statistical significance.

**TABLE 5:** Multivariate linear regression analysis of the relationship among PWMH volume, TWMH volume ipsilateral to the ICAS site, and demographic, clinical, and radiological factors.

**Model**	**Standardization coefficients**	***t***	**Sig.**
	**Beta**		
**PWMH (D)**			
(Constant)		−2.154	0.037
Age	0.544	4.054	**0.000**
**TWMH (D)**			
(Constant)		−0.102	0.919
Age	0.428	3.210	**0.003**
ASPECTS	−0.343	−2.574	**0.014**

*PWMH, peri-ventricular white matter hyperintensities; TWMH, total white matter hyperintensities; ASPECTS, Alberta Stroke Program Early CT score; D, dependent variable. The bold font indicates that the difference achieved statistical significance.*

**FIGURE 4:**
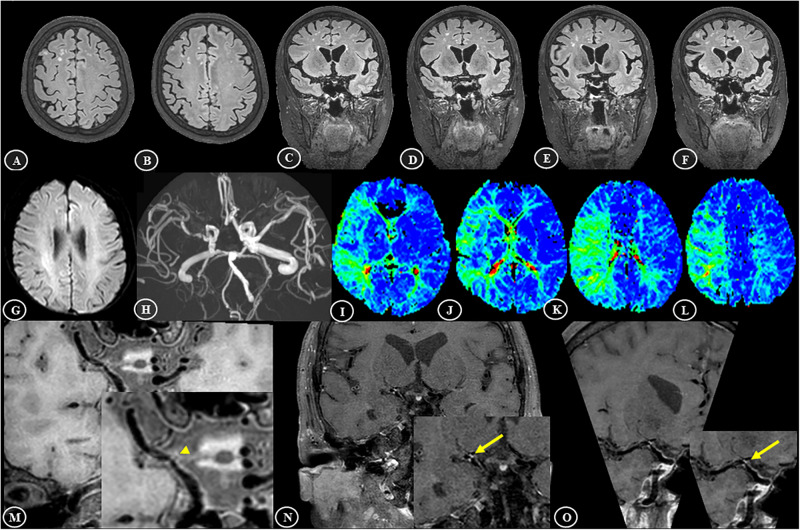
Representative brain MR findings of a 74-year-old female patient with white matter hyperintensities and intracranial atherosclerosis. The patient was admitted with dizziness for 1 month. **(A–F)** On the 3D-FLAIR images, asymmetrically distributed white matter hyperintensities, mainly in the right deep white matter area, are shown. **(G)** Diffusion-weighted imaging (DWI) showed a normal appearance. **(H)** 3D-TOF MRA showed significant stenosis in the M1 segment of right middle cerebral artery (MCA). **(I–L)** According to the Alberta Stroke Program Early CT score (ASPECTS), the TTP maps showed a large area of hypoperfusion, including M1–6 and the insular ribbon, and the TTP-ASPECTS was 3. **(M–O)** T_1_-VISTA image showed an isointense plaque in the right MCA (arrowhead), and the contrast-enhanced T_1_-VISTA image showed strong enhancement of the plaque (grade 2) (arrows).

**FIGURE 5:**
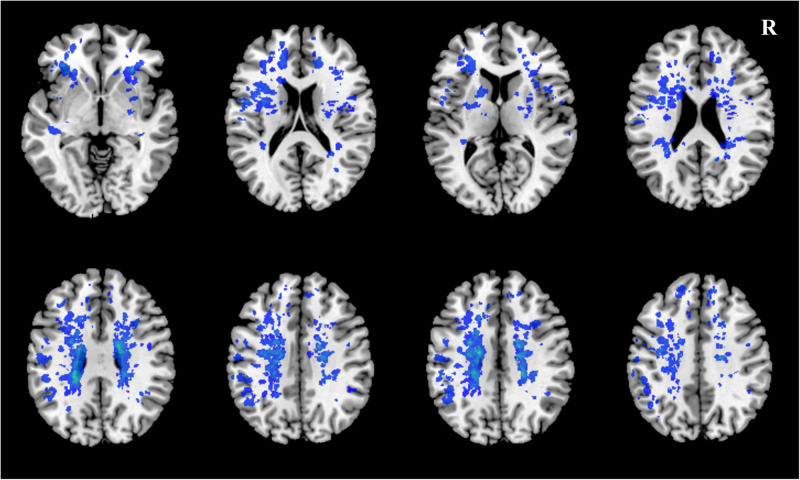
The distribution of WMH lesions in all subjects. All WMH lesions ipsilateral to the ICAS side are shown in the left hemisphere.

A case example demonstrating WMH distribution, plaque characteristics, and TTP-ASPECTS in a patient with dizziness is shown in Figure 4. The distribution of WMH lesions in all subjects is shown in Figure 5, and all the WMH lesions ipsilateral to the ICAS side are shown in the left hemisphere.

### Assessment of Plaque Enhancement, Lumen Stenosis, and TTP-ASPECTS

Our results showed excellent agreement, with kappa values of 0.904 and 0.895 for inter-rater evaluation of plaque enhancement and lumen stenosis, respectively, and an ICC value of 0.915 for TTP-ASPECTS.

## Discussion

In a group of symptomatic unilateral ICAS patients, we found that the ipsilateral DWMH volume was significantly higher than that of the contralateral DWMH volume, while the PWMH volume difference did not reach statistical significance. The inter-hemispheric DWMH volume difference was significantly associated with higher plaque enhancement grade and was inversely associated with TTP-ASPECTS. While age, hypoperfusion, and hypertension were independently associated with ipsilateral DWMH volume, plaque enhancement did not show an association with the ipsilateral DWMH volume.

Our present study focused on assessing the asymmetry of white matter hyperintensities derived from unilateral ICAS patients, which would help to understand the pathogenesis of WMHs. Previous studies comparing the burden of white matter lesions between ipsilateral and contralateral to carotid atherosclerosis stenosis sites and reported conflicting findings. Some studies (Enzinger et al., 2010; Sahin et al., 2015) found a significantly higher load of white matter lesions ipsilateral to carotid atherosclerotic stenosis. However, other studies demonstrated that there was no association between increasing lumen stenosis and ipsilateral WMH volume in patients suffering from either acute infarctions or transient ischemic attack (TIA) by using subjective and/or volumetric analysis of WMHs (Altaf et al., 2008; Potter et al., 2012; Schulz et al., 2013; Wardlaw et al., 2014). These previous studies focused on unilateral carotid stenosis, where cerebral perfusion could be compensated by the contralateral side via the circle of Willis, which would cause a decrease in the asymmetry of WMH volume and thus might result in false-negative results. In our present study, we investigated unilateral ICAS patients and found that the ipsilateral DWMH volume was larger than that of the other non-atherosclerotic site. This observation demonstrates a powerful advantage of the design for comparison between hemispheres, which allows for control of variability among individuals that may otherwise confuse associations between ICAS and WMHs. By using this approach, it can be considered that the impact of atherosclerosis on WMHs is independent of demographic and vascular risk factors, which are shared by small- and large-vessel diseases. Therefore, the asymmetry of WMH volume in our study could be ascribed to unilateral intracranial atherosclerosis.

To date, the pathophysiology of WMHs has not yet been fully elucidated, despite its high prevalence. Coexistence mechanisms (ischemia, disruption of blood–brain barrier, cerebral autoregulation, etc.) may contribute to the development of WMHs (Bridges et al., 2014). An insufficient blood supply due to vascular pathology is thought to be the most important cause of WMHs. Hypoperfusion has been linked with the severity of white matter damage in large samples of individuals (Shi et al., 2016; van Dalen et al., 2016; Chen et al., 2017, 2018; Liu et al., 2018). Low perfusion in normal-appearing white matter (NAWM) has also been associated with subsequent WMH development, and areas of impaired cerebral vascular reactivity (CVR) precede the progression from NAWM to WMH (Sam et al., 2016; Rane et al., 2018), all of which support the role of chronic hemodynamic impairment in the pathogenesis and progression of WMHs. In our study population, age, hypertension, and cerebral hypoperfusion were significantly associated with DWMH severity ipsilateral to the ICAS site among the subjects. Our findings are consistent with previous studies that reported that the prevalence, severity, and progression of WMHs increase with age and hypertension (Lin et al., 2017). Moreover, the hypoperfusion within WMHs may be a direct consequence of extensive local tissue injury and capillary obliteration (Mitchell et al., 2011). The microvascular alterations that are associated with aging (decreased capillary lumen diameter, increased vascular tortuosity, thickened vessel walls, and impaired cerebral autoregulation) and are exacerbated by hypertension (luminal narrowing and vessel wall stiffening) may further impede sufficient perfusion, especially in the distal white matter where the perfusion pressure is the lowest. DWMHs are fed by medullary arteries from the cortical branches of the MCA and are more likely to be sensitive to small-vessel disease (Park et al., 2015). Besides, from the aspect of histopathologic origins, peri-ventricular hyperintensities occur due to venular intramural collagen deposition leading to wall-thickening stenosis (Moody et al., 1995), whereas deep WMHs occur due to arterial insufficiency. Therefore, ICAS can compromise the blood flow of numerous medullary arteries, which might lead to the aggravation of deep white matter perfusion insufficiency and thus may be responsible for the asymmetry of DWMH volume, whereas inter-hemispheric PWMH volume showed no significant difference in our study.

In addition to the abovementioned hemodynamic mechanism, another plausible mechanism for the association between ICAS and WMHs might be arterial–arterial embolism caused by vulnerable plaques. Associations between plaque characteristics and WMH severity ipsilateral to the atherosclerotic site have been reported previously, but the results remain controversial. One study (Altaf et al., 2008) suggested that embolic events from intra-plaque hemorrhage (IPH) within carotid plaques may contribute to the development of ipsilateral WMHs. However, our study found no association between plaque enhancement (a marker of vulnerability to thromboembolism) and ipsilateral DWMH volume among subjects, which is partially in line with the results of earlier studies (Patterson et al., 2009; Kwee et al., 2011) in which the vulnerable plaque was identified by the presence of IPH, lipid-rich necrotic core (LRNC), and fibrous cap (FC) status. Moreover, no associations were found between the presence of carotid IPH, LRNC volume, FC status, and ipsilateral WMH progression over a 1-year period (Kwee et al., 2011). Therefore, no definite causative relationship between atherosclerotic vulnerable plaque and ipsilateral white matter burden can be made based on the existing literature and from our study. Another important finding in our study was that, similar to a study by Altaf et al. (2008), plaque enhancement was significantly associated with the asymmetry of deep white matter lesion volume. Both acute and chronic white matter ischemic lesions may manifest as “WMHs” on T2-weighted or FLAIR images. In addition, studies have shown that acute deep white matter medullary infarcts could lead to the increase in white matter lesions on the images (Lee et al., 2005). In fact, additional focal ischemic white matter injury potentially caused by atherosclerotic stenosis or microemboli from vulnerable plaques cannot be excluded, and we could not differentiate this damage from WMHs, especially in symptomatic patients. Vulnerable plaque is associated with a high risk of occurrence and future recurrence of ischemic events (Wang M. et al., 2017), which is a relatively acute process. The association between the asymmetry of deep white matter lesions and plaque enhancement in our study might suggest that increased deep white matter lesions are those ischemic lesions which are more prone to the development of stroke. While WMHs are commonly attributed to chronic hypoperfusion secondary to atherosclerotic stenosis, this may be the reason why ipsilateral WMH volume is not related to plaque enhancement. Further longitudinal studies that find more acute or chronic infarcts within these regions may help to support this conclusion. Given these results, more attention should be paid when asymmetric white matter lesions are found, and detection of intracranial vessel wall unstable plaques might be necessary to guide treatment strategies.

Our study had several limitations. First, the inherent drawbacks included a relatively small study size and a retrospective study design from a single center, which might limit the statistical power of this study. However, the combination of multiple imaging metrics and vascular risk factors was a strength. Second, the current study utilized a cross-sectional design, which could not evaluate the causality of the association between ICAS and WMHs. A prospective and longitudinal follow-up study would help demonstrate if there was a true association between intracranial disease and deep WMH burden. However, the results of relationships among unilateral ICAS, asymmetry of WMH volume and ipsilateral WMH volume may provide important clues for the direction of future research in this area. Third, because most of the subjects in our study were symptomatic, thus sample selection bias was inevitable. It would be beneficial to have cohorts of symptomatic and asymptomatic patients from multicenters to determine more clearly if the mechanism relates purely to hypoperfusion or other inflammatory mechanisms with an enhancing plaque and to match treatment strategies. Future prospective and longitudinal follow-up studies focused on patients with unilateral atherosclerosis with separate asymptomatic and symptomatic cohorts, standardized medical therapy, and more imaging metrics could provide a better understanding of the link between large-vessel atherosclerosis and white matter disease.

## Conclusion

In conclusion, the DWMH was attributed to chronic hypoperfusion secondary to atherosclerotic stenosis. The association between the asymmetry of deep white matter lesions and plaque enhancement might suggest that increased deep white matter lesions are those ischemic lesions which are more prone to the development of stroke.

## Data Availability Statement

The raw data supporting the conclusions of this article will be made available by the authors, without undue reservation, to any qualified researcher.

## Ethics Statement

The studies involving human participants were reviewed and approved by the Ethics Committee of the Nanjing University Medical School. The patients/participants (or guardian/next of kin where necessary) provided their written informed consent to participate in this study.

## Author Contributions

LN and BZha designed the study. LN, ZQ, and XZ analyzed the imaging data and drafted the manuscript. YX supervised the whole study. FZ and ML collected the data. All authors contributed to the article and approved the submitted version.

## Conflict of Interest

The authors declare that the research was conducted in the absence of any commercial or financial relationships that could be construed as a potential conflict of interest.

## Footnotes

1 www.mricro.com

2 www.itksnap.org
